# Stereotactic ablative radiotherapy for the comprehensive treatment of 4–10 oligometastatic tumors (SABR-COMET-10): study protocol for a randomized phase III trial

**DOI:** 10.1186/s12885-019-5977-6

**Published:** 2019-08-19

**Authors:** David A. Palma, Robert Olson, Stephen Harrow, Rohann J. M. Correa, Famke Schneiders, Cornelis J. A. Haasbeek, George B. Rodrigues, Michael Lock, Brian P. Yaremko, Glenn S. Bauman, Belal Ahmad, Devin Schellenberg, Mitchell Liu, Stewart Gaede, Joanna Laba, Liam Mulroy, Sashendra Senthi, Alexander V. Louie, Anand Swaminath, Anthony Chalmers, Andrew Warner, Ben J. Slotman, Tanja D. de Gruijl, Alison Allan, Suresh Senan

**Affiliations:** 1 Department of Oncology Western University, London Health Sciences Centre, 790 Commissioners Rd. E, London, Ontario N6A4L6 Canada; 2 Department of Radiation Oncology, British Columbia Cancer, Centre for the North, Prince George, BC Canada; 3 Beatson West of Scotland Cancer Centre, Glasgow, UK; 4 Department of Radiation Oncology, Amsterdam UMC Vrije Universiteit Amsterdam Radiation Oncology, Cancer Center Amsterdam, Amsterdam, The Netherlands; 5 Nova Scotia Cancer Centre, Halifax, NS Canada; 6 Alfred Health Radiation Oncology, Melbourne, Australia; 7 Department of Radiation Oncology, Sunnybrook Cancer Centre, Toronto, Canada; 8 Juravinski Cancer Centre, Hamilton, ON Canada; 9 Institute of Cancer Sciences, University of Glasgow, Glasgow, UK

**Keywords:** Oligometastases, Stereotactic radiotherapy, Quality of life, Cancer, Survival

## Abstract

**Background:**

Stereotactic ablative radiotherapy (SABR) has emerged as a new treatment option for patients with oligometastatic disease. SABR delivers precise, high-dose, hypofractionated radiotherapy, and achieves excellent rates of local control for primary tumors or metastases. A recent randomized phase II trial evaluated SABR in a group of patients with a small burden of oligometastatic disease (mostly with 1–3 metastatic lesions), and found that SABR was associated with benefits in progression-free survival and overall survival. The goal of this phase III trial is to assess the impact of SABR in patients with 4–10 metastatic cancer lesions.

**Methods:**

One hundred and fifty-nine patients will be randomized in a 1:2 ratio between the control arm (consisting of standard of care palliative-intent treatments), and the SABR arm (consisting of standard of care treatment + SABR to all sites of known disease). Randomization will be stratified by two factors: histology (Group 1: prostate, breast, or renal; Group 2: all others), and type of pre-specified systemic therapy (Group 1: immunotherapy/targeted; Group 2: cytotoxic; Group 3: observation). SABR is to be completed within 2 weeks, allowing for rapid initiation of systemic therapy. Recommended SABR doses are 20 Gy in 1 fraction, 30 Gy in 3 fractions, or 35 Gy in 5 fractions, chosen to minimize risks of toxicity. The primary endpoint is overall survival, and secondary endpoints include progression-free survival, time to development of new metastatic lesions, quality of life, and toxicity. Translational endpoints include assessment of circulating tumor cells, cell-free DNA, and tumor tissue as prognostic and predictive markers, including assessment of immunological predictors of response and long-term survival.

**Discussion:**

This study will provide an assessment of the impact of SABR on clinical outcomes and quality of life, to determine if long-term survival can be achieved for selected patients with 4–10 oligometastatic lesions.

**Trial registration:**

Clinicaltrials.gov identifier: NCT03721341. Date of registration: October 26, 2018.

**Electronic supplementary material:**

The online version of this article (10.1186/s12885-019-5977-6) contains supplementary material, which is available to authorized users.

## Background

The *oligometastatic state* refers to a stage of disease where a cancer has spread beyond the site of the primary tumor, but is not yet widely metastatic [1]. In patients with a limited oligometastatic burden, emerging evidence suggests that treatment of all sites of disease with ablative therapies (such as surgery or stereotactic radiation) can improve patient outcomes, including overall- and progression-free survival.

Historically, evidence to support the oligometastatic state has consisted of single-arm, non-randomized studies without controls. One classic study reported on over 5000 patients with lung metastases from a variety of primary tumors. In patients who achieved a complete resection of their lung metastases, 5-year overall survival (OS) was 36%, better than might be expected for a cohort of patients with metastatic disease [2]. Similarly, after radiation, a recent pooled analysis of 361 patients with oligometastatic lesions treated with radiation demonstrated a 3-year OS of 56% [3].

It has been suggested the long-term survivals achieved in patients with oligometastases after ablative therapies is merely due to the selection of very fit patients with slow growing tumors, since randomized evidence to support the oligometastatic paradigm has been lacking [4, 5]. However, at least four recent randomized phase II trials now provide some supporting evidence of an oligometastatic state.

### Randomized evidence supporting the oligometastatic state

Two of these four randomized trials were done in the setting of oligometastatic non-small cell lung cancer (NSCLC). In both, patients presented with a primary lung tumor and a limited number of metastatic lesions (1–3 in one trial, 1–5 in the other), and after initial systemic therapy, patients were randomly assigned to standard palliative treatments vs. consolidative ablative treatments to all sites of disease. Both trials were stopped early due to evidence of efficacy, with the ablative treatments achieving a ~ 3-fold improvement in progression-free survival (PFS) [6, 7]. Based on these results, the phase III NRG LU-002 trial is assessing the impact of consolidative ablative therapies on OS.

A third trial, EORTC 40004, examined the impact of an ablative therapy (radiofrequency ablation [RFA]) in patients with colorectal cancer metastatic to the liver. In this trial, patients with a controlled primary tumor and fewer than 10 hepatic metastases not amenable to resection, and with no extra-hepatic disease, were randomized to systemic therapy +/− RFA to all sites of disease [8]. When initially reported [9], the trial showed no difference in OS between arms, but with long-term follow-up (median 9.7 years), a significant difference in OS emerged, with an 8-year OS of 36% in the RFA arm and only 9% in the systemic therapy arm [8].

The fourth trial, Stereotactic Ablative Radiotherapy for the Comprehensive Treatment of Oligometastatic Disease (SABR-COMET) enrolled 99 patients who had controlled primary solid tumors and up to 5 metastatic lesions [10–12]. Patients were randomized in a 1:2 ratio between standard of care (SOC) palliative treatments (Arm 1) vs. SOC + SABR to all sites of disease (Arm 2). The primary endpoint was OS, and the trial employed a randomized phase II screening design, with an alpha of 0.20, in order to provide an initial comparison between arms. More than 90% of patients enrolled had 1–3 metastases. OS was 28 months in Arm 1 and 41 months in Arm 2 (*p* = 0.09), meeting the primary endpoint of the trial. PFS was doubled: 6 months in Arm 1 and 12 months in Arm 2 (*p* = 0.001). SABR was generally well tolerated, with a 29% rate of grade 2 or higher toxicity, although the rate of treatment-related grade 5 toxicity was 4.5%.

Despite this new evidence, many uncertainties remain regarding the oligometastatic state.

### Defining the oligometastatic state

A major unanswered clinical question is the precise definition of the oligometastatic state, namely, how many metastatic lesions are amenable to ablative therapies that may benefit the patient.

Many studies have defined ‘oligometastatic’ as 1–3, or 1–5, metastatic lesions, although some have used broader definitions, including the EORTC 40004 trial described above that allowed up to 9. For example, one single-arm phase II trial in patients with NSCLC enrolled 24 patients with up to 6 active sites of extracranial disease, and treated patients with SABR to all active sites along with erlotinib. The treatment was well-tolerated, with only two grade 3 toxicities. Median OS was 20.4 months, and median PFS was 14.7 months. A second study included NSCLC patients with up to 8 lesions, as long as all could be treated within established dose constraints [13].

In the setting of brain metastases, recent non-randomized evidence suggests that patients may benefit from stereotactic radiotherapy to 4–10 metastatic lesions. The prospective JLGK0901 trial treated 1194 patients who had 1–10 metastatic lesions, with a total cumulative volume of ≤15 mL, and treated all with stereotactic radiosurgery. The study used a non-inferiority design with a primary endpoint was OS, comparing patients with 5–10 lesions vs. those with 2–4. Median OS in both groups was 10.8 months, meeting the primary endpoint of non-inferiority (*p* < 0.0001). Treatment was well-tolerated, with only 9% of patients in either group experiencing adverse events of any grade. A separate retrospective study examined stereotactic radiation in patients with more than 10 brain metastases (where 64% had received prior brain radiotherapy), and concluded that it could be delivered safely, with no episodes of symptomatic necrosis and a 13% rate of radiographic necrosis [14].

The toxicity of SABR may not depend on the overall number of lesions, but moreso the doses delivered to organs at risk. For serial organs, such as the spinal cord, bronchi, and great vessels, reduction of the maximum dose of radiation is expected to reduce the risk of toxicity. For parallel organs, such as the lung, liver and renal cortex, the risk of toxicity may be mitigated by ensuring that a critical volume of the organ is spared from substantial doses of radiation [15]. The typical critical volume to be spared is about 1/3 of the volume of the organ. Therefore, this trial will employ dose constraints for serial structures that ensure minimization of high-dose volumes, constraints for parallel structures that ensure critical volume sparing, and constraints for dose spillage, to ensure that all SABR plans are highly conformal.

The application of ablative therapies for patients with 4–10 metastatic deposits appears promising, based on the encouraging results from randomized trials mostly enrolling patients with 1–3 lesions and the single-arm studies evaluating ablative therapies patients with a larger burden of disease. However, it is likely that as the number of metastases increases, the risk of further distant failure (i.e. development of additional metastases after SABR) will increase, and the risk of toxicity from SABR will likely increase. As a result, the use of SABR in such patients might be best in a scenario where the doses of SABR are lowered to reduce the risk of toxicity, pre-planning of SABR is required before enrollment, and SABR is given immediately prior to systemic therapy that will help to address the risk of occult micrometastases.

In summary, it is unclear if all patients with > 3 oligometastatic lesions benefit from SABR, in terms of improved OS, PFS, or quality of life. The purpose of this randomized trial is to assess the impact of SABR on outcomes in patients with 4–10 oligometastatic lesions.

## Methods/design

The objective of this trial is to assess the impact of SABR, compared to standard of care treatment, on overall survival, oncologic outcomes, and quality of life in patients with a controlled primary tumor and 4–10 metastatic lesions.

### Primary endpoint

- Overall Survival
  - ◦ Defined as time from randomization to death from any cause

### Secondary endpoints

- Progression-free survival
  - ◦ Defined as time from randomization to disease progression at any site or death
- Time to development of new metastatic lesions
- Quality of life
  - ◦ Assessed with the Functional Assessment of Cancer Therapy: General (FACT-G) and the EuroQol - 5 Dimension - 5 Level (EQ-5D-5L)
- Toxicity
  - ◦ Assessed by the National Cancer Institute Common Toxicity Criteria (NCI-CTC) version 4 for each organ treated (e.g. liver, lung, bone)

### Translational endpoints

- Assessment of circulating tumor cells, cell-free DNA, and tumor DNA as prognostic and predictive markers of survival, and for early detection of progression
- Assessment of immunological predictors of response and long-term survival

## Study design

**Fig. 1:**
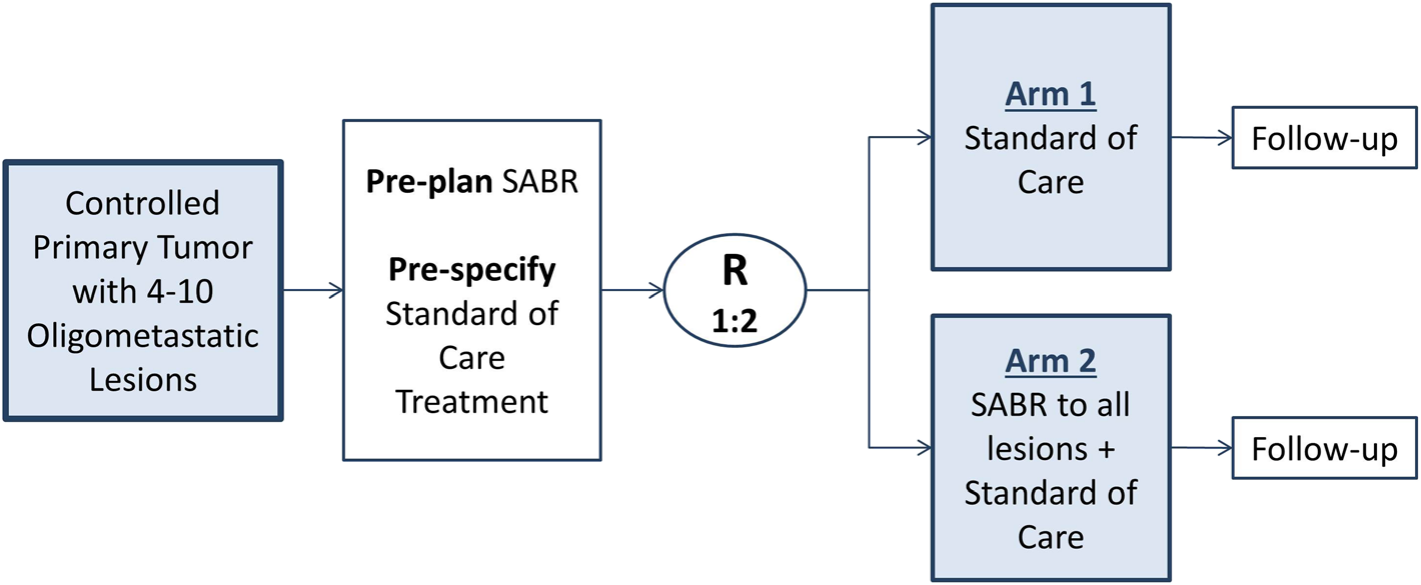
Study Schema

This study is a phase III multicentre randomized trial. Participating centres will be tertiary, academic hospitals or radiotherapy treatment centres in Canada, the United Kingdom, the Netherlands, and Australia (updated country list available on ClinialTrials.gov entry NCT03721341). Patients will be randomized with parallel assignment in a 1:2 ratio between current standard of care treatment (Arm 1) vs. standard of care treatment + SABR (Arm 2) to sites of known disease (Fig. 1).

Patients will be stratified by two of the strongest prognostic factors, based on a large multi-institutional analysis [3]: histology (Group 1: prostate, breast, or renal; Group 2: all others), and type of pre-specified systemic therapy (Group 1: immunotherapy/targeted; Group 2: cytotoxic; Group 3: observation).

## Inclusion criteria

- Age 18 or older
- Willing to provide informed consent
- Karnofsky performance status > 60
- Life expectancy > 6 months
- Histologically confirmed malignancy with metastatic disease detected on imaging. Biopsy of metastasis is preferred, but not required.
- Controlled primary tumor
  - ◦ Defined as at least 3 months since original tumor treated definitively, with no progression at primary site
- Total number of metastases 4–10
- All sites of disease can be safely treated based on a pre-plan

## Exclusion criteria

- Serious medical comorbidities precluding radiotherapy. These include interstitial lung disease in patients requiring thoracic radiation, Crohn’s disease in patients where the gastrointestinal (GI) tract will receive radiotherapy, and connective tissue disorders such as lupus or scleroderma.
- For patients with liver metastases, moderate/severe liver dysfunction (Child Pugh B or C)
- Substantial overlap with a previously treated radiation volume. Prior radiotherapy in general is allowed, as long as the composite plan meets dose constraints herein. For patients treated with radiation previously, biological effective dose calculations should be used to equate previous doses to the tolerance doses listed below. All such cases must be discussed with one of the study PIs.
- Malignant pleural effusion
- Inability to treat all sites of disease
- Any single metastasis > 5 cm in size.
- Any brain metastasis > 3 cm in size or a total volume of brain metastases greater than 30 cc.
- Metastasis in the brainstem
- Clinical or radiologic evidence of spinal cord compression
- Dominant brain metastasis requiring surgical decompression
- Metastatic disease that invades any of the following: GI tract (including esophagus, stomach, small or large bowel), mesenteric lymph nodes, or skin
- Pregnant or lactating women

## Pre-treatment evaluation

### Investigations

- History and Physical Examination
  - ◦ Including prior cancer therapies and concomitant cancer-related medications
- Restaging within 12 weeks prior to randomization:
  - ◦ Brain: CT or MRI for tumor sites with propensity for brain metastasis. All patients with brain metastases (at enrollment or previously treated) require an MRI.
  - ◦ Body: 18-FDG PET/CT imaging is recommended, except for tumors where FDG uptake is not expected (e.g. prostate, renal cell carcinoma). PSMA-PET or choline-PET is recommended for prostate cancer. In situations where a PET scan is unavailable, or for tumors that do not take up radiotracer, CT neck/chest/abdomen/pelvis with bone scan required
  - ◦ Spine: MRI required for patients with vertebral or paraspinal metastases. The MRI needs to image the area being treated and one vertebrae above and below as a minimum, but does not need to be a whole spine MRI unless clinically indicated.
- Liver function tests (AST, ALT, GGT, alkaline phosphatase), albumin, bilirubin, and INR for patients with liver metastases
- Pregnancy test for women of child-bearing age

### Defining the number of metastases

#### Counting Metastases

Patients are eligible if there are 4–10 metastatic lesions present. Each discrete lesion is counted separately. For patients with lymph node metastases, each node is counted as one site of metastasis. All known metastatic lesions must be targetable on planning CT. For patients where the lesion is only detectable on MRI, fusion of the MRI with the planning CT is required. There is no limit to the number of metastases in each individual organ, as long as dose constraints can be met in the pre-plan. For parallel organs such as the liver and lung, patients with several lesions may not meet the pre-plan criteria and therefore will not be randomized.

#### Previously treated metastases

Patients with prior metastases that have been treated with ablative therapies (e.g. SABR, surgery, radiofrequency ablation) are eligible, as long as those metastases are controlled on imaging. In that case, the previously treated lesions are counted toward the total of 10 (e.g. a patient with 3 previous brain metastases treated is allowed to have up to 7 other metastases for enrollment).

If a patient has received systemic therapy and the number of metastases has been reduced, they are eligible for enrollment as long as the total number of metastases prior to systemic therapy was 10 or fewer.

#### Small or indeterminate lesions

When patients have small indeterminate nodules (e.g. a 3 mm lung nodule) it can be difficult to determine whether these are benign or whether they represent metastasis. Any such indeterminate lesion is automatically considered to be a metastasis *unless there are > 2 months of documented stability.* The presence or absence of such indeterminate lesions will be noted on the study enrollment form.

If a lesion is too small to treat due to targeting issues (e.g. a 3 mm lung lesion not likely to be visible on cone beam CT [CBCT]), the following approach is to be taken: if randomized to Arm 1, no intervention is needed, since such a lesion would not require palliative radiation. If randomized to Arm 2, the lesion is followed, and upon progression to a size that is treatable, it should be treated with SABR. This would not be counted as progression.

### Brain metastases at presentation

If a patient presents with 1–3 brain metastases and ablation of those metastases (with surgery or radiation) is judged to be clinically required regardless of the treatment of extracranial metastases, it is permitted. Those treated metastases count within the total number of 10 lesions. The patient would then be randomized to treatment of the extracranial disease or not.

### Patients already receiving systemic therapy

If a patient is already receiving systemic therapy, they are still eligible for enrollment. For example, if a patient with 5 metastases has been on pemetrexed for a year and is planning to continue, they can still be randomized, and if allocated to the standard arm would continue to receive pemetrexed; on the experimental arm SABR would be delivered between cycles, possibly requiring a break in systemic therapy to comply with the timing of systemic therapy described in the Systemic therapy section.

## Interventions

### Standard arm (arm 1)

Radiotherapy for patients in the standard arm should follow the principles of palliative radiotherapy, for the purpose of alleviating symptoms or preventing imminent complications. Recommended dose fractionations in this arm will include 8 Gy in 1 fractions, 20 Gy in 5 fractions, and 30 Gy in 10 fractions. Patients in Arm 1 should not receive stereotactic doses or radiotherapy boosts, unless there is a clearly known clinical benefit (e.g. stereotactic radiation to new brain metastases when all disease is controlled on systemic therapy).

Systemic therapy will be pre-specified based on the standard of care approach for that patient, and it may include systemic therapy (cytotoxic, targeted, hormonal, or immunotherapy) or observation. See Brain metastases at presentation section for the timing of systemic therapy.

### Experimental arm (arm 2)

Stereotactic radiation in Arm 2 will be delivered with three major guiding principles:

- **Minimization of Toxicity**: The SABR doses used herein are lower than those used for radical treatments, and normal tissue tolerance doses will never be exceeded. Concurrent chemotherapy or targeted therapy at the time of radiotherapy is not allowed.
- **Minimization of Treatment Time**. To avoid delays in proceeding to systemic therapy, all SABR will be delivered over the course of 2 weeks.
- **Pre-planning required before enrollment:** To ensure safety, all patients require a pre-plan of their SABR treatments before enrollment. If a patient undergoes pre-planning but cannot be randomized due to failure to generate an acceptable plan, the centre will receive modest compensation to cover pre-planning costs. The baseline information of such patients will be captured (i.e. the Eligibility Checklist and Baseline Form), but they will not be followed for outcomes.

#### Dose/fractionation

Each lesion may be treated with 1, 3, or 5 fractions, depending on the local practice of the enrolling institution and treating physician. All doses are prescribed to the periphery of the planning target volume (PTV).

Acceptable fractionations are listed in Table 1. Three-fraction regimens will deliver a fraction every second day, and five-fraction regimens are delivered daily. All treatments must be completed within 2 weeks (10 working days) in order to avoid delays in starting systemic therapy.

**Table 1:** Allowable doses and fractionations*

Number of Fractions	Preferred Dose	Acceptable Doses	Major Deviation
1	20 Gy	16–24 Gy	< 16 Gy or > 24 Gy
3	30 Gy	24–33 Gy	< 24 Gy or > 33 Gy
5	35 Gy	25–40 Gy	< 25 Gy or > 40 Gy

*Note that centres should use doses that standard at their institutions based on the specific clinical situation, within these guidelines. For example, if the standard dose for a 2.5 cm brain metastasis is 24 Gy in 3 fractions, which is an ‘acceptable dose’, that should be used instead of the ‘preferred dose’

#### Immobilization

Immobilization will be as in the original SABR-COMET trial protocol [11, 12].

#### Imaging/localization/registration

Patients will undergo planning CT simulation with axial CT images obtained throughout the region of interest. For centres using stereotactic radiosurgery platforms, real-time tumor tracking and orthogonal imaging systems are permitted.

Patients treated at the VUmc in Amsterdam may be treated with MRI-guided delivery if deemed appropriate by the treating oncologist, using daily plan adaption as has been described previously [16–19]. The "4D-CT procedures" section will not apply to these patients.

#### 4D-CT procedures

4-dimensional CT will be used for tumors in the lungs, liver, or adrenals. 4D-CT quality assurance procedures are as per the previous SABR-COMET trial [11, 12].

#### Volume definitions (arm 2)

For all lesions, the gross tumor volume (GTV) will be defined as the visible tumor on CT and/or MRI imaging +/− PET. No additional margin will be added for microscopic spread of disease (i.e. Clinical Target Volume [CTV] = GTV). For vertebral body lesions, although some centres consider the entire vertebral body as the CTV, that is not preferred in this trial due to the risk of large cumulative amounts of bone marrow being irradiated. It is strongly preferred that vertebral PTV volumes consist of the GTV (as defined on CT and MRI) with a small margin for motion, and NOT include the whole uninvolved vertebral body. A Planning Target Volume (PTV) margin of 2–5 mm will be added depending on site of disease, immobilization, and institutional set-up accuracy: 2 mm margins should be used for spinal stereotactic treatments, 0–2 mm for brain tumors, and 5 mm for other sites.

Targets should be named based on the organ involved, and numbered cranially to caudally for each organ. For example, in a patient with 1 brain and 3 lung lesions, nomenclature would be: GTV_brain_1, GTV_lung_1, GTV_lung_2, and GTV_lung_3, and corresponding PTV_brain_1, PTV_lung_1, PTV_lung_2_, and PTV_lung_3, representing the lesions from superior to inferior.

For spinal lesions, a pre-treatment MRI is required to assess the extent of disease and position of the cord. This must be fused with the planning CT scan. A Planning Organ at Risk Volume (PRV) expansion of 2 mm will be added to the spinal cord, and dose constraints for the spinal cord apply to this PRV. Alternatively, the thecal sac may be used as the PRV. For radiosurgery platforms, a PRV margin of 1 mm is permitted for the spinal cord.

#### Organ at risk (OAR) doses

OAR doses are listed in Additional file 1. OAR doses may not be exceeded. In cases where the PTV coverage cannot be achieved without exceeding OAR doses, the PTV coverage is to be compromised. All OARs within 5 cm of the PTV must be contoured. This should be tested for each PTV by creating a 5 cm expansion to examine which OARs lie within that expansion.

#### Treatment planning

Treatment can be delivered using static beams (either 3D-conformal radiotherapy or intensity-modulated) or rotational therapy (volumetric modulated arc therapy, or tomotherapy). Priority will be placed on generating clinically acceptable plans while minimizing complexity, planning time, and treatment time.

##### Dose constraints may not be exceeded

If a dose constraint cannot be achieved due to overlap of the target with an organ at risk, the dose can be reduced, the number of fractions can be increased, or the target coverage compromised in order to meet the constraint. The decision as to whether to reduce the dose to the whole target, or part of the target (i.e. by compromising the PTV coverage), is left to the discretion of the treating physician. In cases where the target coverage must be reduced, the priority for dose coverage is the GTV (e.g. attempt to cover as much of the GTV as possible with the prescription dose). For vertebral tumors, note that the spinal cord constraints apply to the PRV (see the "Volume definitions (arm 2)" section).

For all targets, doses should be prescribed to 60–90% isodose line surrounding the PTV, and all hotspots should fall within the GTV. 95% of the PTV should be covered by the prescription dose, and 99% of the PTV should be covered by 90% of the prescription dose.

Doses must be corrected for tissue inhomogeneities. Several non-overlapping 6/10 MV beams (on the order of 7–11 beams) or 1–2 VMAT arcs combined possibly with a few non-coplanar beams should be utilized. Non-coplanar beams can be used to reduce 50% isodose volume.

The number of isocentres is at the discretion of the treating physician, physicists, and dosimetrists. Generally, metastases can be treated with separate isocenters if they are well-separated.

The scheduling and sequence of treating each metastasis is at the discretion of individual physicians, but in general should begin with the brain, due to risks associated with progression. All SABR must be completed within 2 weeks.

#### Quality assurance (arm 2)

In order to ensure patient safety and effective treatment delivery, a robust quality assurance protocol is incorporated. The following requirements must be completed for each patient:

- Prior to treatment, plans for each patient must be peer-reviewed, either by discussion at quality assurance (QA) rounds or by another individual radiation oncologist.
- All radiotherapy plans must meet target dose levels for organs at risk (Additional file 1). Prior to plan approval, the dose to each organ at risk must be verified by the physicist or treating physician.
- All dose delivery for intensity-modulated plans (including arc-based treatments) will be confirmed before treatment by physics staff.

#### Systemic therapy

Patients treated with prior systemic therapy are eligible for this study, however, systemic therapy agents that are cytotoxic, immunotherapeutic, or molecularly targeted agents are NOT allowed within the period of time commencing 2 weeks prior to radiation lasting until 1 week after the last fraction. Hormone therapy is exempted from this and is allowed during treatment. Use of chemotherapy schemes containing potent enhancers of radiation damage (e.g. gemcitabine, doxorubicin) are discouraged within the first month after radiation.

#### Concurrent steroid treatment for brain metastases

Patients who require systemic steroids as treatment for brain metastases or related edema should be tapered as quickly and as safely possible. Prolonged use of steroids should be avoided, and steroid use will be recorded.

#### Further radiotherapy for progressive disease at new metastatic sites

Patients in Arm 1 who develop new, untreated metastatic deposits should be treated with standard-of-care approaches. SABR to those sites is not permitted, except for unique scenarios where it would be considered standard of care (e.g. all disease controlled on systemic therapy with a newly developed brain metastasis).

Patients in Arm 2 who develop new, untreated metastatic deposits should be considered for SABR at those sites, as appropriate, if such deposits can be treated safely with SABR, and if the treating institution offers SABR for that body site. If SABR is not possible, then palliative RT can be delivered if indicated. Patients in Arm 2 who develop progression at lesion previously treated with SABR may be considered for palliative radiation or repeat SABR if safe and dose constraints can be met.

#### Quality assurance for centres joining study

Prior to opening the study, each participating research centre will be required to send to one of the Principal Investigators a mock treatment plan for the anatomic sites that will be treated (e.g. lung, brain, liver, adrenal), to ensure that the treatment plans are designed in compliance with the protocol. The principal investigators will provide pertinent CT datasets. Alternatively, a pre-plan for a patient enrolled on this trial may be used for credentialing. Each participating research centre can choose which tumor sites will be treated at their individual centre (i.e. some centres may only choose to treat a subset of the eligible metastatic sites). Sites that have prior accreditation for SABR through a clinical trial (e.g. SABR-COMET, or organ-specific SABR trials) are exempt from this requirement for the organ sites that have been accredited in those trials.

## Subject discontinuation / withdrawal

Subjects may voluntarily discontinue participation in the study at any time. If a subject is removed from the study, the clinical and laboratory evaluations that would have been performed at the end of the study should be obtained. If a subject is removed because of an adverse event, they should remain under medical observation as long as deemed appropriate by the treating physician.

## Follow-up evaluation and assessment of efficacy

### Follow-up Prior to Progression

Patients will be seen every 3 months post-randomization for the first 2 years, and every 6 months until 5 years after treatment (Table 2). At each visit, a history and physical examination will be conducted by the oncologist, and CTC-AE toxicities recorded. The FACT-G and EQ-5D-5 L quality of life questionnaire is to be completed at each visit.

**Table 2:** Follow-up Evaluations

Test and Procedures	1–4 weeks post SABR treatment and prior to systemic therapy	3 Months post Randomization	Years 1–2	Years 3–5	First progression or study completion (at 5 years post-randomization) whichever is first
			Every 3 months	Every 6 months	
History and Physical including assessment of side affects			X	X	
CT or MR head, CT chest, abdomen, pelvis			X	X	
Bone Scan			X	X	
Completion of questionnaires (FACT-G and EQ-5D-5 L)			X	X	
Blood Samples for Correlative Studies (i.e studies that are associated with the main study)	X (Arm 2)	X (Arm 1 & 2)			X (Arm 1 & 2)

CT head (or MR head), CT chest, abdomen and pelvis, and bone scans will be repeated every 3 months for the first 2 years, then every 6 months until 5 years have elapsed. Head imaging can be omitted for histologies without a propensity for brain metastases (e.g. prostate), and bone scans may be omitted in patients without bone metastases at presentation. PET scanning may be used in follow-up for patients who were staged with a PET scan for trial entry. In such cases, the PET replaces the CTs of the chest, abdomen, pelvis and the bone scan; brain imaging would still be required for histologies with a propensity for brain metastases.

Since many patients will be receiving systemic therapy and separately-timed imaging may be required to assess response to systemic therapy, attempts should be made to avoid duplication of scans. The imaging requirements herein may be adjusted by ±4 weeks in order to align with scans used to assess response to systemic therapy.

### Follow-up after progression

After progression, patients randomized to Arm 2 will be considered for salvage SABR if new sites of disease develop, as long as it can be delivered safely, and to a maximum of 10 lesions total (including lesions treated at baseline).

After progression, for patients in either arm, additional visits, imaging or laboratory investigations should be carried out at the discretion of the oncologist. Additional treatment (e.g. further systemic therapy) is at the discretion of the treating oncologists. However, additional treatments, toxicities of study treatment, vital status and quality of life should still be collected, along with any further anti-cancer treatment delivered (e.g. further palliative radiation or systemic therapy), and this may be ascertained remotely (e.g. by phone or mail) to minimize visit burden for patients.

### Assessment of efficacy

- Overall Survival
  - ◦ Defined as time from randomization to death from any cause
- Progression-free survival
  - ◦ Defined as time from randomization to disease progression at any site or death.
  - ◦ Progression is defined as per the Response Evaluation Criteria in Solid Tumors (RECIST) 1.1 guidelines (http://recist.eortc.org/recist-1-1-2/). It can be difficult to distinguish recurrence from fibrosis/pseudoprogression after stereotactic radiation in some locations, such as the lung or brain. In such cases, if the RECIST 1.1 criteria for progression are met, the situation should be counted as progression unless there is imaging follow-up with stability of the imaging findings for at least 6 months.
  - ◦ As per RECIST 1.1, when findings of progression are equivocal (e.g. small new lesions of uncertain etiology), the patient should still be followed. If progression is confirmed at the next assessment, the date of progression assigned is the earlier date when progression was first suspected.
- Time to development of new metastatic lesions
  - ◦ Defined as the time from randomization to the development of new lesions that were not detectable at the time of randomization. In a situation where indeterminate lesions were present at randomization, progression at one of those lesions does not count as a new metastatic lesion.
  - ◦ As noted above, as per RECIST 1.1, when findings of new metastases are equivocal (e.g. small new lesions of uncertain etiology), the patient should still be followed. If progression is confirmed at the next assessment, the date of progression assigned is the earlier date when progression was first suspected.
- Quality of life
  - ◦ Assessed with the Functional Assessment of Cancer Therapy: General (FACT-G) and the EQ-5D-5 L
- Toxicity
  - ◦ Assessed by the National Cancer Institute Common Toxicity Criteria (NCI-CTC) version 4 for each organ treated (e.g. liver, lung, bone)]

## Statistical considerations

### Randomization

The study will employ a 1:2 randomization between Arm 1: Arm 2, based on the stratification factors described in Methods/design section. Patients will be randomized in permuted blocks, with the size of the blocks known only to the statistician. The randomization sequence is known only to the statistician and uploaded into a restricted-access database (REDCap) housed on secure hospital servers at LHSC. Upon enrollment of a patient, the database will be accessed by the trial co-ordinator to obtain the next intervention in the random sequence, for the pertinent stratum, which will then be assigned to the patient.

### Sample size calculation

The sample size calculation is based on the OS results in the SABR-COMET trial. Overall, in that trial, median OS was 28 months in the standard arm and 41 months in the SABR arm. In the patients with 4–5 metastases, median OS in that trial was 7 months in the standard arm and 14 months in the SABR arm. These latter numbers are not reliable, since the number of patients with 4–5 metastases was very small, but they are useful to illustrate that the expected OS will decrease as the number of lesions increases.

In this current trial, we hypothesize that the median OS will be 10 months in Arm 1 and 17 months in Arm 2. In order to detect this difference, with an alpha of 0.05, 80% power, and a 5% dropout rate, 159 patients will be required. The study projects accrual over 60 months with 12 months of additional follow-up. Analysis will take place at least 12 months after the last patient is accrued, once 122 total OS events have occurred.

### Analysis plan

Patients will be analyzed in the groups to which they are assigned (intention-to-treat). De-identified data (except for study number and initials, see confidentiality below) will be transmitted from participating centres via REDCap to be collected centrally where it will be stored on secure hospital servers at LHSC. Source documents will also be uploaded. Research coordinators (clinical trials staff) will perform data checks throughout the trial period and will call participating centres or visit as necessary. PFS and OS will be calculated using the Kaplan-Meier method with differences compared using the stratified log-rank test. Pre-planned subgroup analyses will occur based on the stratification factors. A Cox multivariable regression analysis will be used to determine baseline factors predictive of survival endpoints. For the endpoint of time to new metastases, a Fine and Gray competing risk analysis will be used to account for competing risk of death. Quality of life at 6 months will be measured using FACT-G and EQ-5D-5 L scores, with differences between groups tested using the Student’s t-test. Differences in rates of grade 2 or higher toxicity between groups will be tested using the Fisher’s Exact Test or Chi-Squared test, as appropriate.

### Data safety monitoring committee

The DSMC membership will be independent of the study sponsor and free of competing interests. The DSMC will meet annually after study initiation to review toxicity outcomes. If any grade 3–5 toxicity is reported, the DSMC will review the case notes to determine if such toxicity is related to treatment. If the DSMC deems that toxicity rates are excessive (> 40% grade 3 toxicity, or > 8% grade 5 toxicity), then the DSMC can, at its discretion, recommend cessation of the trial, dose adjustment, or exclusion of certain treatment sites and/or delivery techniques that are deemed as high-risk for complications.

### Interim analysis

The DSMC will conduct one interim analysis once the 75th patient is accrued and followed for 6 months. For this interim analysis, the DSMC will be blinded to the identity of each treatment arm, but median OS data will be presented for each arm. The DSMC will recommend stopping the trial if there is an OS difference that is statistically significant with a threshold of *p* < 0.001 using the stratified log-rank test.

It is deemed worthwhile to stop for futility if both the OS and PFS analyses are likely to be negative. Therefore, at this interim analysis, if the hazard ratios for OS and PFS in Arm 2 vs Arm 1 are BOTH > 1.0 (i.e. a higher hazard rate for OS in the experimental arm, and a higher hazard rate for PFS in the experimental arm) using univariable Cox regression, then the trial will be stopped for futility.

### Future pooled analysis with SABR-COMET-3

A separate but similar phase III trial, but for patients 1–3 metastases, called SABR-COMET-3, is being proposed and drafted at the same time as this current trial. Once both trials are complete, a separate pooled analysis, using individual patient data from both trials, will be conducted, with the primary endpoint of OS, and any of the secondary endpoints from either trial where data has been collected in both trials.

## Biomarker studies

**Fig. 2:**
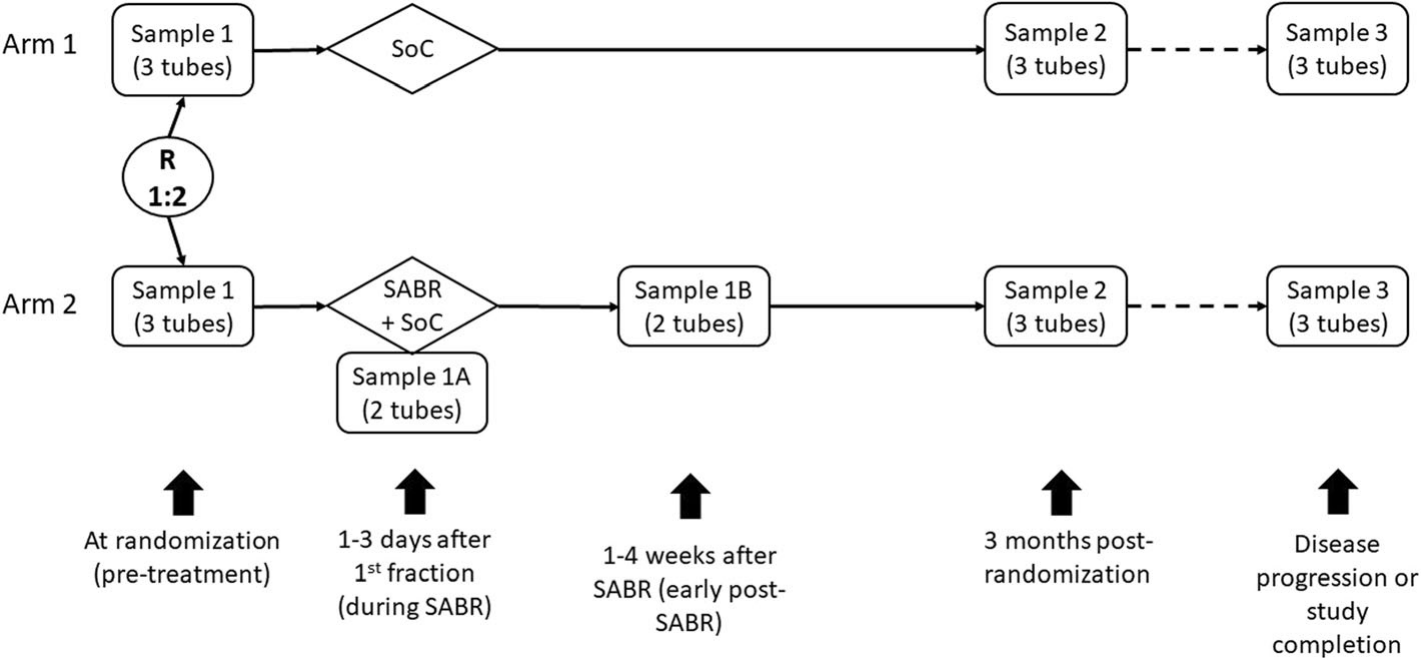
Peripheral Blood Collection Timeline. Study completion is defined as 5 years of follow-up. Sample 1A & 1B will include 2 vials of blood for ctDNA and peripheral blood mononuclear cell isolation

The mandatory translational component of this trial has been designed to minimize the impact on patients while addressing important research questions around the oligometastatic state. Specifically, the increased requirements, beyond standard of care testing, consist of drawing 3 tubes of blood at 3 time periods (Fig. 2) for all patients: at randomization, 3-months post-randomization, and at progression.

Patients in Arm 2 only also require a blood draw 1–3 days after their first fraction of SABR and then 1–4 weeks after completion of SABR, prior to systemic therapy. In patients who do not progress, the final sample will be drawn at study completion (5-years post randomization).

Blood sample collection must take place on Monday, Tuesday, Wednesday or Thursday such that biospecimens requiring immediate overnight shipping (CTCs) can be received and processed quickly, without samples sitting unprocessed over a weekend.

Specimens 1A and 1B are required only for patients in Arm 2 and consist of 2 tubes of blood for ctDNA (plasma) and peripheral blood mononuclear cells (PBMCs). The timing of these blood draws can be chosen to minimize visits for the patient, but must occur within 1–3 days after the 1st fraction of SABR and 1–4 weeks post-SABR (prior to systemic therapy), respectively. For example, since many patients will likely be receiving multiple single-fraction treatments over > 1 day or will be receiving 3- or 5-fraction SABR, the 1A blood draw can be completed upon a patient’s return for the second SABR treatment. This blood draw is preferred to occur on the day after the first fraction. Similarly, since many patients will be proceeding to systemic therapy within a few weeks of SABR, completing the 1B blood draw on the first day of systemic therapy, but prior to the delivery of the systemic therapy, would be reasonable.

In addition to blood samples, the study will collect tissue samples from previous biopsies or resections of the primary tumor and metastases, where available. No additional biopsies will be needed for the purposes of the biomarker component of this trial beyond those collected as part of routine clinical care. If formalin-fixed paraffin-embedded (FFPE) tissue blocks are not available to be sent at the discretion of the local pathologist (e.g. insufficient tissue, or a need to keep all tissue for future purposes), the FFPE tissue blocks are not required and centres can proceed by sending peripheral blood only.

### Laboratory support and shipping

Each participating institution will require an on-site laboratory for peripheral blood sample processing. This laboratory must also have freezer storage (− 80 °C and liquid nitrogen). The protocols for collection and processing of peripheral blood, including required equipment and reagents, are provided in the Laboratory Manual. All shipping costs will be covered via the provision of pre-paid shipping labels, and an additional small stipend will be provided to cover laboratory time. A ‘biomarker studies kit’ containing collection tubes and pre-paid shipping labels will be sent to each participating institution to be retained by the personnel responsible for biospecimen collection.

### Translational studies: Background & Rationale

At the present time, there are no biomarkers that define the oligometastatic state. The closest to a defining biological feature is tumor histology, of which breast, kidney, and prostate are associated with improved OS in patients with clinical oligometastatic disease [3]. Key clinical characteristics - colloquially termed ‘The Four Aces’ [20] - help to identify a patient sub-population with metastatic cancer that is most likely to benefit from ablation of all sites of disease.

Even within this group, however, outcome can be variable: while some patients exhibit long disease-free intervals and better-than-expected overall survival following ablation of metastases, others progress rapidly and extensively with poor survival outcomes [21]. Elucidating the biological mediators underlying a more indolent, sequential pattern of progression (i.e., oligometastasis) versus rapid, “poly-metastatic” progression will allow for more accurate selection of patients whose intrinsic natural history of disease make them more likely to benefit from ablation.

Specific biological characteristics of oligometastatic disease could provide important predictive biomarkers in this setting, but have thus far remained elusive. Studies up to this point have focused on micro-RNA profiling, but unfortunately these studies have not identified an miRNA expression signature that consistently defines patients with few metastases [22–24]. No other studies to our knowledge have sought to identify specific biomarkers of oligometastasis. While a wide array of pre-clinical analyses have identified genetic and epigenetic alterations associated with metastasis in general [25], it remains to be determined which of these features represent useful biomarkers in differentiating rapid and widely metastatic cancer from an oligometastatic natural history.

### Translational studies: purpose

To assess the correlation between candidate biomarkers of oligometastatic disease (blood- or tissue-derived) and oncologic outcomes including response to SABR, disease progression, and overall survival.

### Methodology: the liquid biopsy

To evaluate potential biomarkers in a clinical setting, the use of a “liquid biopsy” is less invasive and more practical alternative to repeat biopsies. A liquid biopsy refers to sampling of peripheral blood to isolate and characterize circulating tumor DNA (ctDNA), circulating tumor cells (CTCs), and/or circulating host immune cells, among others [26]. Liquid biopsy is an ideal sampling technique in this clinical trial because biopsy of metastatic lesions is not always possible, and unlike metastectomy, SABR does not inherently yield tissue. Moreover, there is evidence that post-SABR anti-tumor immune activation can be detected in the peripheral blood [27] and that tumor necrosis (the immunogenic cell death mechanism associated with SABR) is associated with greater ctDNA concentrations [28], thus making liquid biopsy a rational means by which to assess potential biomarkers longitudinally.

Despite its many potential advantages, liquid biopsy does have some drawbacks, including the fact that discordance has been observed between genotyping via ctDNA versus tumor tissue; however, this may merely reflect clonal or temporal heterogeneity [29]. Thus, for genetic analysis, we propose a combined approach that capitalizes on published findings of large-scale whole-genome sequencing efforts (including multi-region sequencing studies) [25, 28, 30, 31] to inform: (a) targeted panel-based evaluation of FFPE tumor tissue (primary tumor and/or metastasis biopsy) to assess genetic loci that are most frequently altered in metastatic disease (mutation or copy-number variation), followed by; (b) downstream analysis of peripheral blood that is tailored to detect tumor genetic alterations previously detected in (a) or more broadly assessed for copy number alteration and mutation (panel-based approach).

## Circulating tumor cell analysis

Circulating tumor cells have repeatedly demonstrated their utility as a clinical prognostic metric. Prospective clinical studies have provided evidence that CTCs are prognostic in metastatic breast, prostate, and colorectal cancer, whereby increasing concentration correlates with oncologic outcomes such as treatment response and survival [26]. Recently, the largest pooled CTC analysis to date revealed that CTC enumeration identifies an indolent subgroup of metastatic breast cancer patients (Stage IV_indolent_) with improved survival, independent of treatment or molecular subtype [32]. The role of CTCs in oligometastatic disease has not been studied, yet the sub-population with slowly-progressing natural history may overlap with the clinical definition of oligometastasis. Thus, CTCs may represent a useful prognostic and/or predictive biomarker and their evaluation in this setting is warranted.

### Analysis

A peripheral blood sample will be collected at each participating institution into provided CellSave blood collection tubes (Menarini Silicon Biosystems; preferred) or Cell-Free DNA BCT® blood collection tubes (Streck) which stabilize CTCs for 96 h at room temperature. Samples will then be prepared for CTC analysis using the CellSearch system (Veridex, Inc.) in the laboratory of Dr. Alison Allan. Participating institutions will ship samples within 24 h to LHSC for processing and CellSearch analysis.

## Host immune cell analysis

The role of the host immune system in establishing a prohibitive or permissive microenvironment for metastatic colonization is increasingly well-established: while activation of cytotoxic T-lymphocytes is thought to inhibit metastases, regulatory T-lymphocytes can conversely exhaust/de-activate anti-tumor immunity, thus having the opposite effect [33]. Additionally, recent evidence suggests that natural killer cells contribute to non-specific immune surveillance to create an inhospitable milieu for the establishment of metastatic colonies [34]. Furthermore, as evidenced by the successful application of immune-checkpoint inhibition in treating metastatic cancer, the modulation of the host immune system can dramatically impact the extent of metastasis. Finally, both pre-clinical and clinical data demonstrate that immune cell activity can also be modulated by SABR [35], an effect that can be monitored in peripheral blood via analysis of circulating immune cells following radiotherapy [27]. SABR may also effect a so-called abscopal (out-of-field) response, thereby improving control of metastatic disease [36]. Given the important role of immune surveillance for metastasis, its therapeutic modulation in the setting of metastatic disease, and its interplay with SABR, evaluating the importance of host immunity in the context of oligometastasis ablation is warranted to explore useful predictive and/or prognostic biomarkers.

### Analysis

We aim to analyze peripherally circulating immune cells for expression of surface antigens that are reflective of immune activation or exhaustion/suppression. The analysis itself will be conducted by the Amsterdam UMC. Samples will be collected and stored at each participating institution as per the Laboratory Manual and stored at − 80 °C will subsequently be shipped, on an annual basis, on dry ice to VU Amsterdam for further processing and FACS analysis.

## Tumor DNA analysis

Recent studies utilizing contemporary genomic analysis techniques have identified individual gene-level alterations (e.g., mutations and copy-number variations) as well as genome-scale metrics (e.g., tumor mutational burden and percent genomic copy-number alteration) that correlate with metastatic disease and poor outcomes [25, 28, 30, 31]. Perhaps most informatively, multi-region sequencing of primary tumors and paired metastases has permitted phylogenetic analysis of metastasis evolution, shedding light on genetic alterations that correlate with patterns of metastatic dissemination; specifically, select genetic alterations in the setting of renal cancer effectively differentiate a rapid, multi-site “poly-metastatic” progression from an attenuated, indolent metastatic disease course reminiscent of an oligometastatic natural history [30]. These large-scale studies have performed whole-genome sequencing in each patient, an approach that is not currently practical or cost-effective in the clinical setting. However, curating the findings of these large-scale analyses to develop a targeted approach using a panel-based subset of frequently-altered genetic loci will permit a more focused yet rationally-based evaluation of oligometastatic tumor DNA, akin to the approach taken by Abbosh et al. [28].

### Analysis

The plasma fraction containing circulating tumor DNA (ctDNA) will be isolated from peripheral blood collected at the above-mentioned timepoints as per the protocol detailed in the Laboratory Manual. Once extracted, plasma samples will be frozen at -80 C. Participating institutions will batch-ship frozen samples on dry ice to LHSC where all samples will be stored.

## Confidentiality

The names and personal information of study participants will be held in strict confidence. All study records (case report forms, safety reports, correspondence, etc.) will only identify the subject by initials and the assigned study identification number. The investigator will maintain a confidential subject identification list (Master List) during the course of the study. Access to confidential information (i.e., source documents and patient records) is only permitted for direct subject management and for those involved in monitoring the conduct of the study (i.e., Sponsors, CRO’s, representatives of the IRB/REB, and regulatory agencies). The subject’s name will not be used in any public report of the study.

## Data sharing statement

Deidentified participant data from this trial will not be shared publicly, however, the full protocol will be published along with the primary analysis of the outcomes.

## Protocol ammendments and trial publication

Any modifications to the trial protocol must be approved and enacted by the principal investigator (Current version: 1.0 on January 31, 2018). Protocol amendments will communicated to all participating centres, investigators, IRBs, and trial registries by the principal investigator. Any communication or publication of trial results will be led by the principal investigator, and is expected to occur within 1 year of the primary analysis. Trial results will remain embargoed until conference presentation of an abstract or until information release is authorized. Authorship of the trial abstract and ultimately the full manuscript will be decided by the principal investigator at the time of submission. Professional writers will not be used for either abstract or manuscript preparation.

## Discussion

The oligometastatic paradigm posits the existence of an intermediate state between localized and widely-disseminated metastatic cancer [1]. In this setting, resection or ablative therapy to metastases is associated with better-than-expected survival [2, 3]. Recent randomized data have helped to confirm the existence of the oligometastatic state and demonstrate that ablative therapy - including stereotactic ablative radiotherapy (SABR) - improves progression-free and overall survival [6, 7]. These studies are based mostly on patients with 3 or fewer metastases; while SABR is generally safe, the risk of treatment-related toxicity is expected to rise with the number of metastases treated. Therefore, considerable equipoise remains as to whether patients with a greater number of metastases would similarly benefit from SABR to all sites. It is incumbent upon physicians to determine how many lesions are amenable to safe, minimally-toxic ablative therapy that benefits the oligometastatic patient. Furthermore, a practical definition of oligometastatic disease will be aided by a deeper understanding of its biological underpinnings, which thus far have remained elusive [22–24]. Identifying biomarkers associated with a relatively indolent, sequential pattern of progression (i.e., oligometastasis) will thus facilitate greater accuracy in selecting which oligometastatic patients are most likely to benefit from SABR.

The SABR-COMET-10 trial is a multicenter, international phase III trial that aims to accrue 159 patients with 4–10 metastases, randomized to standard of care versus standard of care plus SABR to all metastatic lesions. The delivery of SABR in this trial will be guided by key principles including pre-planning prior to enrolment to ensure safety, SABR dose reduction and strict adherence to OAR tolerances to minimize toxicity, and treatment completion within 2 weeks to prevent delay in systemic therapy initiation/resumption. The primary endpoint of SABR-COMET-10 is OS with secondary endpoints of PFS and QoL. Translational endpoints will also be assessed using peripheral blood samples collected at multiple timepoints to evaluate circulating tumour DNA, circulating tumour cells, and host immune cell activation. Thus, SABR-COMET-10 aims to determine both whether SABR improves outcomes in patients with > 3 metastases as well as to identify biomarkers of oligometastasis that can help select those patients who are most likely to benefit.

This trial has several important limitations. Inclusion of all histologies allows for more rapid accrual and reduces the risk of failure due to poor enrollment, but will not allow us to elucidate differences in outcomes by histologic subtype. In addition, our estimate of a 10-month survival in the control arm is based on the results of the original SABR-COMET trial, wherein patients with 4–5 metastases in the control arm had a median survival of only 7 months. We inflated this estimate to 10 months, to increase our power to detect a difference if outcomes have improved based on improvements in standard of care systemic therapy since the original trial. If the true survival in the standard arm is substantially longer, then statistical power might be reduced. As in the original SABR-COMET trial, in SABR-COMET-10 there is no specified limit to the number of lesions that can be treated with palliative local treatments (such as external beam radiation) on the standard arm. Ablative treatments are not expected to be provided in Arm 1, unless considered standard of care (e.g. stereotactic radiation for brain metastases), and all such treatments delivered will be documented. We are optimistic that the pragmatic components of this trial, the large number of participating centres, and the presence of physician equipoise on this question will help to reduce the risk of poor accrual.

## Additional files

Additional file 1: Dose Constraints. Dose Constraints for Treatment Planning. (DOC 123 kb)

Additional file 2: World Health Organization Trial Registration Dataset. List of Fields in Trial Registration Database. (DOC 76 kb)

Additional file 3: Sample Consent Form. Sample Consent Form. (DOC 129 kb)

## Data Availability

Not applicable.

## Abbreviations

18-FDG: 18-Fluorodeoxyglucose
4D-CT: Four-Dimensional Computed Tomography
ALT: Alanine Aminotransferase
AST: Aspartate Aminotransferase
CBCT: Cone Beam Computed Tomography
CT: Computed Tomography
CTC: Circulating Tumor Cells
CTC-AE: Common Toxicty Criteria for Adverse Events
ctDNA: Circulating Tumor Deoxyribonucleic Acid
CTV: Clinical Target Volume
DNA: Deoxyribonucleic Acid
DSMC: Data Safety Monitoring Committee
EORTC: European Organization for Research and Treatment of Cancer
FACT-G: Functional Assessment of Cancer Therapy: General
FFPE: Formalin-Fixed Parafin-Embedded
GGT: Gamma Glutamyl Transpeptidase
GI: Gastrointestinal
GTV: Gross Tumour Volume
IGTV: Internal Gross Tumor Volume
INR: International Normalized Ratio
LHSC: London Health Sciences Centre
miRNA: Micro-Ribonucleic Acid
MRI: Magnetic Resonance Imaging
NCI-CTC: National Cancer Institute Common Toxicity Criteria
NSCLC: Non-Small Cell Lung Cancer
OS: Overall Survival
PBMC: Peripheral Blood Mononuclear Cells
PET: Positron Emisson Tomography
PFS: Progression-Free Survival
PRV: Planning Organ at Risk Volume
PSMA: Prostate Specific Membrane Antigen
PTV: Planning Target Volume
QA: Quality Assurance
RECIST: Response Evaluation Criteria in Solid Tumors
RFA: Radiofrequency Ablation
RNA: Ribonucleic Acid
SABR: Stereotactic Ablative Radiotherapy
SABR-COMET: Stereotactic Ablative Radiotherapy for the Comprehensive Treatment of Oligometastatic Disease
SOC: Standard of Care
VMAT: Volumetric Modulated Arc Therapy
VUmc: Vrije University Medical Center
