# Supplementary material for: Stereotactic ablative radiotherapy for the comprehensive treatment of 4–10 oligometastatic tumors (SABR-COMET-10): study protocol for a randomized phase III trial
Source: BMC Cancer. 2019 Aug 19;19:816. doi: 10.1186/s12885-019-5977-6 (PMC6699121; doi:10.1186/s12885-019-5977-6)
Supplement: Supplementary file 1 — Dose Constraints. Dose Constraints for Treatment Planning. (DOC 123 kb) [file 12885_2019_5977_MOESM1_ESM.doc]

# Additional file 1: Dose Constraints

These are based on the NRG-LU002 trial ) and the SABR-COMET trial. If any structure is not listed, the constraints may be calculated using the linear quadratic formula from accepted QUANTEC doses, using an alpha-beta ratio of 2 for late effects.

**Table A1: Dose Constraints for Serial Structures.** D0.03cc = maximum dose in Gy allowable to the hottest 0.03 cc; other D values are used in the same way

| **Structure** | **Volume** | **1 Fraction** | **3 Fraction** | **5 Fraction** |
| --- | --- | --- | --- | --- |
| **Optic Pathway** | D0.03cc | 10 | 17.4 | 25 |
|  | D0.2cc | 8 | 15.3 | 23 |
| **Cochlea** | D0.03cc | 9 | 14.4 | 22 |
| **Brainstem** | D0.03cc | 15 | 23.1 | 31 |
|  | D0.5cc | 10 | 15.9 | 23 |
| **Spinal Cord** | D0.03cc | 14 | 22.5 | 28 |
|  | D0.35cc | 10 | 15.9 | 22 |
| **Cauda Equina or Sacral Plexus** | D0.03cc | 16 | 22.5 | 31.5 |
|  | D5cc | 14 | 21.9 | 30 |
| **Esophagus** | D0.03cc | 15.4 | 25.2 | 35 |
|  | D5cc | 11.9 | 17.7 | 19.5 |
| **Brachial Plexus** | D0.03cc | 16.4 | 26 | 32.5 |
|  | D3cc | 13.6 | 22 | 27 |
| **Heart** | D0.03cc | 22 | 30 | 38 |
|  | D15cc | 16 | 24 | 32 |
| **Great Vessels** | D0.03cc | 37 | 45 | 53 |
|  | D10cc | 31 | 39 | 47 |
| **Trachea and large bronchi** (mainstem, bronchus intermedius) | D0.03cc | 20.2 | 30 | 40 |
|  | D4cc | 17.4 | --- | --- |
|  | D5cc | --- | 25.8 | 32 |
| **Chest Wall or Rib** | D0.03cc | 33 | 50 | 57 |
|  | D5cc | 28 | 40 | 45 |
| **Skin** | D0.03cc | 27.5 | 33 | 38.5 |
|  | D10cc | 25.5 | 31 | 36.5 |
| **Stomach** | D0.03cc | 22 | 30 | 35 |
|  | D10cc | 17.4 | 22.5 | 26.5 |
| **Bile Duct** | D0.03cc | 30 | 36 | 41 |
| **Duodenum** | D0.03cc | 17 | 22.2 | 26 |
|  | D5cc | 11.2 | 15.6 | 18.5 |
|  | D10cc | 9 | 12.9 | 14.5 |
| **Jejunum or Ileum** | D0.03cc | 22 | 27 | 32 |
|  | D30cc | 12.5 | 17.4 | 20 |
| **Colon** | D0.03cc | 29.2 | 34.5 | 40 |
|  | D20cc | 18 | 24 | 28.5 |
| **Rectum** | D0.03cc | 44.2 | 49.5 | 55 |
|  | D3.5cc | 39 | 45 | 50 |
|  | D20cc | 22 | 27.5 | 32.5 |
| **Ureter** | D0.03cc | 35 | 40 | 45 |
| **Bladder** | D0.03cc | 25 | 33 | 38 |
|  | D15cc | 12 | 17 | 20 |
| **Penile Bulb** | D3cc | 16 | 25 | 30 |
| **Femoral Heads** | D10cc | 15 | 24 | 30 |

**Table A2: Dose Constraints for Parallel Structures.** Parallel structures require the use of a ‘critical volume’ (CV), also termed a ‘complementary volume’. For example, for lung, the CV1500cc is listed as 7 Gy for 1-fraction trevalue atments, meaning that there must be 1500 cc of lung receiving 7 Gy or less. This is read from the left-hand side of a DVH. For further information on calculating the CV, with DVH examples, see *Application of Critical Volume-Dose Constraints for Stereotactic Body Radiation Therapy in NRG Radiation Therapy Trials* . VX refers to the percent of lung (minus GTVs) receiving X Gy or more.

| **Structure** | **Volume** | **1 Fraction** | **3 Fraction** | **5 Fraction** |
| --- | --- | --- | --- | --- |
| **Lung (combined right and left, subtract GTVs)** | CV1500cc | 7 | 10.5 | 12.5 |
|  | V8Gy(%) | 37 |  |  |
|  | V11Gy(%) |  | 37 |  |
|  | V13.5Gy(%) |  |  | 37 |
| **Liver** | CV700cc | 11 | 17.1 | 21 |
| **Kidney cortex (combined left and right)** | CV200cc | 9.5 | 15 | 18 |

**Table A3: Constraints for Dose Spillage and Conformality**

The R100 (ratio of size of prescription isodose volume to size of PTV) should be less than 1.2. Exceptions are allowed for small PTVs. The R50 (ratio of size of 50% prescription isodose volume to size of PTV) and the maximum dose 2 cm or more away from the PTV (D2cm) should conform to the requirements of NRG-LU002 (see protocol for details )

**References**

1. **NRG-LU002 - "Maintenance Systemic Therapy Versus Local Consolidative Therapy (LCT) plus Maintenance Systemic Therapy for Limited Metastatic Non-Small Cell Lung Cancer (NSCLC): A Randomized Phase II/III Trial."**. *Protocol available at CTSUorg; password required*.

2. Ritter TA, Matuszak M, Chetty IJ, Mayo CS, Wu J, Iyengar P, Weldon M, Robinson C, Xiao Y, Timmerman RD: **Application of Critical Volume-Dose Constraints for Stereotactic Body Radiation Therapy in NRG Radiation Therapy Trials**. *International Journal of Radiation Oncology • Biology • Physics* 2017, **98**(1):34-36.
