# Supplementary material for: Stereotactic ablative radiotherapy for the comprehensive treatment of 4–10 oligometastatic tumors (SABR-COMET-10): study protocol for a randomized phase III trial
Source: BMC Cancer. 2019 Aug 19;19:816. doi: 10.1186/s12885-019-5977-6 (PMC6699121; doi:10.1186/s12885-019-5977-6)
Supplement: Supplementary file 2 — World Health Organization Trial Registration Dataset. List of Fields in Trial Registration Database. (DOC 76 kb) [file 12885_2019_5977_MOESM2_ESM.doc]

# Additional file 2: World Health Organization Trial Registration Dataset

| **Item** | **Description** |
| --- | --- |
| Primary registry and trial identifying number | ClinicalTrials.gov NCT03721341 |
| Date of registration in primary registry | October 26, 2018 |
| Secondary identifying numbers | NA |
| Source(s) of monetary or material support | London Health Sciences Foundation (philanthropic donations) and Ontario Institute of Cancer Research |
| Primary sponsor | Dr. David A. Palma |
| Secondary sponsor(s) | NA |
| Contact for public queries | Dr. David A. Palma |
| Contact for scientific queries | Dr. David A. Palma |
| Public title | High-dose, precision radiation therapy to treat all sites of metastatic disease in patients with 4-10 metastases |
| Scientific title | Stereotactic Ablative Radiotherapy for Comprehensive Treatment of 4-10 Oligometastatic Tumors (SABR-COMET 10) |
| Countries of recruitment | Canada, United Kingdom, The Netherlands, |
| Health condition(s) or problem(s) studied | Metastatic cancer |
| Intervention(s) | Standard Arm: Standard of care treatment: palliative radiotherapy, chemotherapy, immunotherapy, hormones, or observation, is at the discretion of the treating oncologist. |
|  | Stereotactic Arm: Stereotactic ablative radiotherapy, plus standard of care treatment: chemotherapy, immunotherapy, hormones, or observation given at the discretion of the treating oncologist. |
| Key inclusion and exclusion criteria | Inclusion: Age 18 or older; willing to provide informed consent; Karnofsky performance score greater than 60; Life expectancy greater than 6 months; Histologically confirmed malignancy with metastatic disease detected on imaging; Biopsy of metastasis is preferred, but not required; Controlled primary tumor defined as at least 3 months since original tumor treated definitively with no progression at primary site; Total number of metastases 4-10; All sites of disease can be safely treated based on a pre-plan |
|  | Exclusion: Serious medical comorbidities precluding radiotherapy; For patients with liver metastases, moderate/severe liver dysfunction (Child Pugh B or C); Substantial overlap with a previously treated radiation volume; Prior radiotherapy in general is allowed, as long as the composite plan meets dose constraints herein; For patients treated with radiation previously, biological effective dose calculations should be used; Malignant pleural effusion; Inability to treat all sites of disease; Any single metastasis greater than 5 cm in size; Any brain metastasis greater than 3 cm in size or a total volume of brain metastases greater than 30 cc; Metastasis in the brainstem; Clinical or radiologic evidence of spinal cord compression; Dominant brain metastasis requiring surgical decompression; Metastatic disease that invades any of the following: GI tract (including esophagus, stomach, small or large bowel), mesenteric lymph nodes, or skin; Pregnant or lactating women |
| Study type | Randomized by permuted blocks sequence |
|  | No masking/blinding (open label) |
|  | Parallel assignment |
|  |  |
| Date of first enrolment | February 2019 (anticipated) |
| Target sample size | 159 |
| Recruitment status | Not yet recruiting |
| Primary outcome(s) | Overall survival |
| Key secondary outcomes | Progression-free survival; Time from randomization to development of new metastasis; Quality of Life; Toxicity; Translational endpoints |
