# Supplementary material for: Stereotactic ablative radiotherapy for the comprehensive treatment of 4–10 oligometastatic tumors (SABR-COMET-10): study protocol for a randomized phase III trial
Source: BMC Cancer. 2019 Aug 19;19:816. doi: 10.1186/s12885-019-5977-6 (PMC6699121; doi:10.1186/s12885-019-5977-6)
Supplement: Supplementary file 3 — Sample Consent Form. Sample Consent Form. (DOC 129 kb) [file 12885_2019_5977_MOESM3_ESM.doc]

# Additional file 3: Consent Form

**study information and Informed consent form**

***Stereotactic Radiation to Treat Patients with 4-10 Locations of Cancer Spread***

**A Randomized Phase III Trial of Stereotactic Ablative Radiotherapy for the Comprehensive Treatment of 4-10 Oligometastatic Tumors (SABR-COMET 10)**

Study ID: SABR-COMET 10

Study Doctor: Dr. ________________

*If an REB approved French consent is not used at your institution remove this statement.*

Le formulaire de consentement est disponible en français sur demande.

**Emergency Contact Number** (24 hours / 7 days a week): _________________________

Non-Emergency contact numbers are at the end of this document under Contacts.

**Introduction**

You are being invited to participate in a clinical trial (a type of study that involves research). Clinical trials only include participants who choose to take part. You are invited to participate in this trial because you have cancer that has spread to other parts of your body (metastatic cancer). When cancer has spread to only a few locations, this is called oligo-metastatic disease.

This consent form provides you with information to help you make an informed choice. Please read this document carefully and take your time in making your decision. You may find it helpful to discuss it with your friends and family.

Taking part in this study is voluntary. You may choose not to take part or if you choose to participate may leave the study at any time without giving a reason. Deciding not to take part or deciding to leave the study later will not result in any penalty or any loss of benefits to which you are entitled.

**Background**

The usual treatment for your disease is radiation therapy and/or drug therapy (such as chemotherapy or hormone therapy), or sometimes observation, depending on your type of cancer.

This research is being done because the current usual treatments for oligo-metastatic disease have been unable to get rid of the cancer altogether. Stereotactic ablative radiotherapy (SABR) is a new radiation treatment that delivers high-dose, precise radiation to small tumors in 1-3 weeks of treatment. This new technique can potentially allow radiation treatments to be focused more precisely, and be delivered more accurately than with older treatments. This improvement could help by reducing side effects overall (through radiation exposure to a smaller area of the body and over a shorter time period) and by improving the chance of controlling the cancer by more precisely treating the cancer and by giving higher doses of radiation. SABR is considered a standard treatment for small lung cancers, and selected cancers that have spread to the brain, but its value for patients with oligo-metastatic cancer is not known.

The research ethics board, which oversees the ethical conduct of research involving humans, has reviewed and accepted this study.

**Purpose**

The purpose of this study is to compare the effects on you and your cancer of SABR*,* compared to the commonly-used approaches to treat this disease. The commonly-used approaches are called ‘standard of care’ treatment, and could include drug treatments (such as hormone therapy or chemotherapy), radiation to treat symptoms, or observation.

The study will compare how long patients live after each treatment, rates of side effects, and quality of life.

**Alternative Treatments**

You do not have to take part in this study in order to receive treatment/care. Other options (in addition to the standard or usual treatment described above) may include, but are not limited to:

- No therapy at this time.
- Alternative chemotherapy agents or other drug treatments like immunotherapy or targeted treatments, if they are appropriate for your type of cancer.
- Palliative care or Best Supportive Care (BSC). This type of care helps reduce pain, tiredness, appetite problems and other problems caused by the cancer. It does not treat the cancer directly, but instead tries to improve how you feel. Best Supportive Care tries to keep you as active and comfortable as possible.
- Other experimental studies may be available if you do not take part in this study.

Please talk to your study doctor or usual cancer doctor about the known benefits and risks of these other options before you decide to take part in this study. Your usual cancer doctor can also discuss with you what will happen if you decide not to undertake any treatment at this time.

**Expected Number of Participants**

About 159 people from Canada, the Netherlands, and other countries will take part in this study. The study will take about 6 years to complete and the results should be known in about 8 years.

Your study doctor will be informed of the results of this study once they are known.

**Assignment to a Group**

If you decide to participate you will be "randomized" into one of the groups described below. Randomization means that you are put into a group by chance. It is like flipping a coin. There is no way to predict which group you will be assigned to. You will have a 2 in 3 chance of being placed in the group receiving SABR. Neither you nor your doctor can choose what group you will be in.

You will be told which treatment you are to get.

**Group 1: (NON-EXPERIMENTAL TREATMENT**): Standard of Care Arm (this may include chemotherapy, hormonal therapy, immunotherapy or observation).

Your study doctors will tell you what the standard treatment for your cancer is before you join the study, and this is the treatment you will receive if you are assigned to Group 1.

If you are randomized to Group 1 you will receive standard palliative radiation therapy to any symptomatic sites. You may receive chemotherapy, hormonal, immunotherapy therapy at the discretion of your study doctor.

Standard Palliative Radiation Therapy

You will receive palliative radiation therapy to any symptomatic sites (locations that are causing problems). This will involve a CT scan to design the radiation treatment, followed by between one and ten daily treatments of low-dose radiation to improve symptoms.

**Group 2: (EXPERIMENTAL TREATMENT)**: SABR followed by standard of care treatment (e.g. chemotherapy, hormonal therapy, or immunotherapy)

If you are randomized to Group 2 you will receive stereotactic ablative radiotherapy to all sites of metastatic cancer.

After the SABR is finished, you will still receive the standard of care treatment, except for the palliative radiation therapy, since all sites of cancer will receive SABR radiation.

The radiation planning process may involve construction of a plastic mask or special bean-bag to hold your head or body still for treatment followed by a CT scan. The information from the CT scan will be used to target the tumor and minimize the dose to normal tissues.

Treatments will be given either daily or every other day, on weekdays, over 1-2 weeks, depending on the location of your metastatic cancer. The procedure will take about 1 hour. A CT scan through the region being treated will be taken on the radiation unit prior to treatment each day and your position for the treatment adjusted if necessary. Once your positioning is confirmed, the treatment will be given.

The doses of radiation used in Arm 2 are higher than in Arm 1, since the goal of treatment on Arm 2 is to kill all the cancer cells, rather than just improve symptoms. As a result, the risk of side effects will be higher in Arm 2 than in Arm 1 (see below for a description of potential side effects).

After the SABR is finished, you may still receive chemotherapy or targeted therapy, at the discretion of your study doctor.

Chemotherapy

Chemotherapy or other drugs can be given to participants on Arm 1 or Arm 2, starting two weeks or later after the SABR is done. Chemotherapy can consist of drugs that are given either by mouth or intravenously (given into one of your veins by needle), and in some cases can slow the growth of cancer. The decision as to whether you will receive chemotherapy will be made together by you and your medical oncologist. If you are assigned to Group 1, you will receive the standard of care chemotherapy or drug treatment (potentially after some palliative radiation, if needed), In Group 2, you would first receive SABR before the standard of care chemotherapy or drug treatment.

**Study Procedures**

Please see the table at the end of this section.

**Non-Experimental Procedures**

The following tests will be done as part of this study. Some of these tests may be done as part of your standard care, in which case the results may be used. Some of these tests may be done more frequently than if you were not taking part in this study and some may be done solely for the purpose of the study. If the results show that you are not able to continue participating, your study doctor will let you know:

- blood tests – Liver function tests for participants with liver metastases
- history and physical examination
- pregnancy test for women of child-bearing potential
- computed tomography (CT) scan or magnetic resonance imaging (MRI) of metastatic disease sites – A CT scan is a series of x-rays of the body from many angles that are turned into 3-dimensional pictures on a screen. CT scans often involve injecting a dye into your vein. A MRI is an imaging technique that uses a strong magnet to produce pictures of areas inside the body. MRI is useful for assessing organs and other soft tissue, such as the inside of bones.
- Full body 18-FDG/PET scan: A PET scan is used to try to find small deposits of cancer that can’t be detected on CT scans, using a special radioactive sugar that is called 18-FDG. It involves injecting a small amount radioactive sugar into your vein, and this sugar is taken in by cancer cells. The location of the sugar in the body tells your doctor where cancer cells might be. This may not be required for this clinical study.

bone scan – a type of imaging procedure that involves injecting dye into your vein and then taking a series of pictures of all of your bone;

**Questionnaires:**

You will be provided with questionnaires before starting this study, and then every 3 months after start of treatment for the first two years, then every 6 months until 5 years after start of treatment. The purpose of the questionnaires is to understand how your treatment and illness affects your quality of life. These questionnaires ask about how you are feeling and take about 10 minutes to complete.

The information you provide is for research purposes only and will remain strictly confidential. Some of the questions are personal; you can decide not to answer these if you wish.

Even though you may have provided information on the questionnaires, these responses will not be reviewed by your health care team or study team. If you wish them to know this information please bring it to their attention.

**Mandatory Sample Collection**

The researchers doing this study are interested in doing additional research (described below) to evaluate outcomes including response to SABR, disease progression and overall survival.

The purpose of this sample collection is to see we can detect parts of the cancer (either cancer cells or DNA from the cancer) in participants’ blood, and use that to help us learn when a cancer is more likely to come back in the future. We are also looking at markers of the immune system in the blood, to see if those can tell us which cancers are more likely to come back. Samples from your cancer will also be tested to see if certain genes can tell us when a cancer is more likely to come back. You do not need any extra biopsies or tissue samples in this study.

Hereditary genetic testing (to find out if cancer runs in your family) will not be done on these samples.

The collection of these samples is a necessary part of this study and will be used only for these purposes. The samples will not be sold. Once these tests have been completed, any leftover samples will be returned to the facility from which they were obtained if needed or destroyed. If you participate in this study it is possible that not enough tumour tissue will be left for other testing that may need to be done in the future. Please speak to your study doctor to discuss this possibility.

Certain types of genetic testing could have implications for you or your biological relatives.  The researchers believe the chance these things will happen is very small, but cannot promise that they will not occur.

Please ask your study doctor whether this might apply to you as a result of your participation in this study.

Reports about any research tests done with your samples will not be given to you or your study doctor. These reports will not be put in your medical records.

If you are a First Nations or an indigenous person who has contact with spiritual Elders, you may want to talk to them before you make a decision about this research study. Elders may have concerns about some research procedures including genetic testing.

**Tissue Collection (Required)**

A small sample of your tumour that has already been removed by a previous surgery or biopsy will be obtained by the researchers doing this study. No further surgeries or biopsies are required of you for this purpose. If your biopsy or surgery were completed at another institution, signing this consent form means that you are consenting to the collection of your tissue sample, together with any related personal health information, from that institution. You may still participate if a sample is not available.

These tissue samples will be sent to a laboratory at PaLM Core Facility at London Health Sciences Centre, London, Ontario Canada.

**Blood Collection (Required)**

Blood samples will be taken by inserting a needle into a vein in your arm. These will be taken at the same time as your study related tests whenever possible. 3-4 tubes of blood (20 mL, or 4 teaspoons) will be taken with a needle from a vein in your arm at the following times depending on which group you have been assigned to.

- ARM 1: Blood will be drawn at 3 time points: at randomization, 3-months post-randomization, and at progression or after 5-years of follow-up, whichever occurs first.
- ARM 2: Blood will be drawn at 5 time points at randomization, within 1-3 days post SABR, 1-4 weeks post SABR, 3-months post-randomization, and at progression or after 5-years of follow-up, whichever occurs first.

Blood samples will be sent to a laboratory at the Amsterdam University Medical Center, location VU University Medical Center.

**Identification of Samples**

To protect your identity, the information that will be on your samples will be limited to your participant study code, date and time sample was drawn.

**Withdrawal of Required Samples**

If you no longer want your samples to be used in this research, you should tell your study doctor. Your study doctor will notify the sponsor, who will ensure the samples are returned to the hospital from which they were obtained if needed, or destroyed. If tests have already been done on your sample(s) it will not be possible to withdraw those results. However, no further testing will be done. You may still participate in the main study if you withdraw these samples.

**Summary of Treatments, Tests and Procedures**

PRE-TREATMENT EVALUATION

| Test and Procedures | Up to 12 weeks prior to randomization | At Randomization |
| --- | --- | --- |
| History and Physical  (Including prior cancer therapies and concomitant cancer-related medications and assessment of side affects) | X |  |
| CT or MR head, MRI Spinal | X |  |
| 18-FDG PET/CT or  CT neck/chest/abdomen/pelvis with bone scan | X |  |
| Liver function tests for patients with liver metastases | X |  |
| Pregnancy test (for women of childbearing potential) | X |  |
| Completion of questionnaires (FACT-G and EQ-5D-5L ) |  | X |
| Blood Samples for Correlative Studies (i.e studies that are associated with the main study) |  | X(Arm1&2) |

**Study Treatment**

| Test and Procedures | Day 1 | Start Radiation Treatment Approximately 14 Days After Randomization | 1-3 days after 1^st^ fraction of SABR |
| --- | --- | --- | --- |
| Planning CT to design radiation treatment | X |  |  |
| Start Radiation Treatment* |  | X |  |
| Blood for Correlative Studies (i.e studies that are associated with the main study) |  |  | X(Arm2) |

* Radiation schedule will depend on sites of tumor being treated, but generally every day or every other day for 1-2 weeks.

**Follow-up Evaluations**

| Test and Procedures | 1-4 weeks post SABR treatment and prior to systemic therapy | 3 Months post Randomization | **Years 1-2** | **Years 3-5** | First progression or study completion (at 5 years post-randomization) whichever is first |
| --- | --- | --- | --- | --- | --- |
|  |  |  | Every 3 months | Every 6 months |  |
| History and Physical including assessment of side affects |  |  | X | X |  |
| CT or MR head, CT chest, abdomen, pelvis |  |  | X | X |  |
| Bone Scan |  |  | X | X |  |
| Completion of questionnaires (FACT-G and EQ-5D-5L) |  |  | X | X |  |
| Blood Samples for Correlative Studies (i.e studies that are associated with the main study) | X  (Arm 2) | X (Arm1&2) |  |  | X  (Arm1&2) |

**Responsibilities**

If you choose to participate in this study, you will be expected to:

- Tell your study doctor about your current medical conditions;
- Tell your study doctor about all prescription and non-prescription medications and supplements, including vitamins and herbals, and check with your study doctor before starting, stopping or changing any of these. This is for your safety as these may interact with the treatment you receive on this study;
- Tell your study doctor if you are thinking about participating on another research study;
- Return any questionnaires that were completed to the clinic/hospital;
- Tell your study doctor if you become pregnant or father a child while participating on this study

**Length of Participation**

Your treatment with either standard of care with palliative radiation therapy or standard of care with SABR will last for about 1 to 2 weeks, depending on the location of your metastases. You will be asked to come back to the clinic/hospital every 3 months for the first 2 years, then every 6 months for 3-5 years.

You may be seen more often if your study doctor determines that this is necessary, or if your cancer gets worse*.*

No matter which group you are randomized to, and even if you stop treatment early, we would like to keep track of your health for the next five years to look at the long-term effects of the study treatments. This would be done by phone contact or during your follow up visits every 3-6 months.

**Early End to Participation**

The researchers can take you off the study treatment early for reasons such as:

- The treatment does not work for you and your cancer comes back or gets worse.
- You are unable to tolerate the study treatment
- You no longer wish to participate.
- You are unable to complete all required study procedures
- New information shows that the study treatment is no longer in your best interest.
- Your study doctor no longer feels this is the best treatment for you.
- The Sponsor decides to stop the study
- The Research Ethics Board withdraws permission for the study to continue
- You become pregnant

If your participation in the study is stopped your study doctor will provide information about how to stop safely.

**Risks of Participation**

Participating in this study will put you at risk for the side effects listed below. You should discuss these with your study doctor. As with any treatment additional unexpected and sometimes serious side effects are a possibility.

Your study doctor will watch you closely to see if you have side effects. When possible other drugs will be given to you to make side effects less serious and more tolerable. Many side effects go away shortly after treatment is stopped but in some cases side effects can be serious, long-lasting, permanent, or even cause death.

If you experience serious side effects that require treatment between regular clinic/hospital visits, it is important that you make every effort to return to the clinic/hospital where your study treatment was given. If you need immediate treatment and are unable to return to the clinic/hospital where you received your study treatment, you should go to the nearest medical clinic/hospital and tell them that your study doctor should be contacted as soon as possible.

If you require palliative radiation on the Standard of Care Arm (Group 1) or are on the SABR arm (Group 2), you will undergo a CT scan, called a “simulation”, to design the radiation. This CT scan is considered standard for radiation treatment and exposes you to a small amount of radiation. Some patients find the CT scanning table uncomfortable, and in some cases a mild painkiller (e.g. Tylenol) is given for those patients. For the SABR arm, the CT scan will take longer. **Risks and side effects related to radiation therapy depend on the area being treated**. For example, for a patient receiving radiation to the brain, the side effects related to the lungs and bowels do not apply.

Risks and side effects related to radiation are listed below. All risks are expected to be higher with SABR than with the lower-dose treatments.

**Very likely [Common] (*21% or more, or higher than a 1 in 5 risk)*:**

- Fatigue
- Skin Rash in area being treated
- Hair loss in area being treated

**Less likely [*Occasional*] (*5 to 20% or between a 1 in 5 and 1 in 20 risk*):**

- Nausea/vomiting
- Decreased hearing or irritation of the ears
- Dryness or irritation of the eyes
- Dry or sore mouth or throat or loss of taste during radiation treatments.
- Temporary lung injury resulting in shortness of breath or cough (if the lungs are being treated)
- Temporary difficulty or painful swallowing
- Diarrhea or cramping of the bowels (if the abdomen or pelvis are treated)
- Discomfort or frequency of urination (if the pelvis is treated)

**Rarely (*1 to 4% or between a 1 in 25 and 1 in 100 risk*):**

- Permanent lung injury resulting in shortness of breath or cough (if the lungs are being treated)
- Bone injury resulting in a broken bone (if a bone is treated)
- Changes in thinking or memory (if the brain is treated).
- Persistent cramping, diarrhea or bleeding from the bowel (if the abdomen or pelvis is treated)
- Persistent frequency or discomfort with urination
- Persistent pain in a bone, muscle, or nerve

**Rare but Serious (less than 1% or less than a 1 in 100 risk)**

Radiation treatments are associated with a small risk of serious injury to tissues or organs that are included in the area being treated. This injury may show up months to years post treatment. In very rare instances, these side effects may result in death. Some of these side effects include (depending on whether these areas are being treated):

- - Brain injury resulting in loss of strength, sensation or thinking ability
  - Spinal cord injury resulting in paralysis of the lower half of the body including both legs
  - Esophagus injury resulting in difficulty swallowing
  - Heart injury resulting in a heart attack or fluid collection around the heart
  - Rectal or bowel injury resulting in bleeding or perforation (hole in the lining of the bowel and/or rectum) or fistula (abnormal connection between the bowel and another organ)
  - Bladder injury resulting in bleeding or perforation (hole in the lining of the bladder or fistula (abnormal connection between the bladder and another organ)
  - Development of a second cancer in the radiation area, usually several years after treatment.

Your study doctor will monitor your therapy and make adjustments to your treatment or prescribe medicines in order to manage side effects that occur during treatment. The radiation technique, daily dose and total dose of radiation for your treatment will be prescribed by your study doctor in order to minimize the chance of late serious injury as outlined above.

It is possible, although unlikely, that SABR may be associated with unexpected side effects that are not yet known or included on this list. For example, when SABR was first implemented, treatment of tumors in the center of the chest was associated with a high risk of injury to the breathing passages (bronchi), and so the doses delivered to tumors in that area have been lowered to reduce that risk.

The risks and side-effects of the standard or usual treatment will be explained to you as part of your standard care and are therefore not listed.

It is possible that other drugs (prescription and non-prescription drugs), vitamins, or herbals can interact with the radiation used in this study. This can result in either the radiation not working as expected or result in severe side effects.

**Reproductive Risks**

Radiation therapy may be harmful to an unborn baby (fetus). You must not become pregnant or father a baby while receiving radiation therapy and for 6 months after the last dose. Your study doctor will discuss methods with you to ensure you do not become pregnant or father a baby during the study.

Women should not nurse (breastfeed) a baby while receiving radiation therapy and for 6 months after the last dose because the radiation used in this study might be present in breast milk and could be harmful to a baby.

If you become pregnant or father a child during this study or for 6 months after you stop receiving radiation therapy, then you should immediately notify your study doctor. Your study doctor will let the sponsor know about the pregnancy.

If you become pregnant, the researchers or sponsor for this study will access information on the outcome of the pregnancy (the child’s health etc.). This information will be gathered from your medical/study record. This may also involve contacting you for the next year to ask about the health of your child. The researchers or sponsor also may ask to contact the child’s father to get information related to the pregnancy. If you become pregnant and do not want the researchers/sponsor to collect this information, you must let your study doctor know.

If you father a child, the researchers or sponsor for this study will ask to contact the child’s mother to collect information on the outcome of the pregnancy (the child’s health etc.). The child’s mother will be given a separate consent document to sign to give permission for the collection of this information, if a pregnancy should happen.

The child’s mother may choose not to give consent for the collection of this information or may withdraw their consent at any time without giving a reason. This will not impact your participation on the study and will not result in any penalty or any loss of benefits to which you are entitled.

**Data Safety Monitoring Board/Committee**

A Data Safety Monitoring Board/Committee, an independent group of experts, will be reviewing the data from this research throughout the study.

**Benefits**

If you agree to take part in this study, the experimental treatment may or may not be of direct benefit to you. Your cancer may shrink but these things cannot be predicted for you. The researchers hope the information learned from this study will help other patients in the future.

**Confidentiality**

Records identifying you at this centre will be kept confidential and, to the extent permitted by the applicable laws, will not be disclosed or made publicly available, except as described by this consent document.

Studies involving humans now routinely collect information on race and ethnicity as well as other characteristics of individuals because these characteristics may influence how people respond to different medications. Providing information on your race or ethnic origin is voluntary.

Authorized representatives of the following organizations may look at your original (identifiable) medical/clinical study records at the site where these records are held, for quality assurance (to check that the information collected for the study is correct and follows proper laws and guidelines):

- Lawson Health Research Institute, the sponsor of this study
- The Ontario Cancer Research Ethics Board, which oversees the ethical conduct of this study in your clinic/hospital;

Authorized representatives of the above organizations and the organizations listed below may **receive** information related to the study from your medical/clinical study records for quality assurance and data analysis. Your name or other information that may identify you will not be used. The records used by these organizations may contain your participant code, partial initials, sex and partial date of birth.

- PaLM Core Facility at London Health Sciences Centre, London, Ontario Canada.
- Amsterdam University Medical Center, location VU University Medical Center

All of the organizations listed in the above confidentiality sections are required to have strict policies and procedures to keep the information they see or receive about you confidential, except where disclosure may be required by law. The study doctor will ensure that any personal health information collected for this study is kept in a secure and confidential location as required by law. There are federal and provincial laws that these organizations must comply with to protect your privacy.

If the results of this study are published, your identity will remain confidential. It is expected that the information collected during this study will be used in analyses and will be published/presented to the scientific community at meetings and in journals.

Even though the likelihood that someone may identify you from the study data is very small, it can never be completely eliminated.

A copy of this signed and dated consent form may be included in your health record/hospital chart.

Your family doctor/health care provider will be informed that you are taking part in a study so that you can be provided with appropriate medical care. If you do not want your family doctor/health care provider to be informed, please discuss with your study doctor.

A wallet card will be provided to you with information about how to contact the study staff when required.

Your de-identified data from this study may be used for other research studies. If your study data is shared with other researchers, information that links your study data directly to you will not be shared.

**Registration of Clinical Trials**

A description of this clinical trial will be available on http://www.clinicaltrials.gov. This website will not include information that can identify you. At most, the Web site will include a summary of the results. You can search this website at any time.

**Costs**

The study treatment will be provided to you free of charge while you are participating in this study. The costs of your medical treatment will be paid for by your provincial medical plan to the extent that such coverage is available. There may be extra costs that are not covered by your medical plan that you will have to pay yourself; some examples may be physiotherapy or certain pain medications.

Taking part in this study may result in added costs to you (i.e. transportation, parking meals, or unpaid leave from work). You may have to pay for medication prescribed to treat or prevent side effects, and you may have to visit the hospital more often than if you were not participating in this study.

**Compensation**

You will not be paid for taking part in this study.

It is possible that the research conducted using your samples and/or the study data may eventually lead to the development of new diagnostic tests, new drugs or other commercial products. There are no plans to provide payment to you if this happens.

In the case of research-related side effects or injury, medical care will be provided by your doctor or you will be referred for appropriate medical care.

**Rights**

You will be told, in a timely manner, about new information that may be relevant to your willingness to stay in this study.

If you decide to stop participating in the study or if your participation has been stopped, your doctor will discuss other options with you and continue to treat you with the best means available.

You may withdraw your permission to use your personal health information for this study at any time by letting the study doctor know. However, this would also mean that you withdraw from the study. Your study data that was recorded before you withdrew will be used but no information will be collected or sent to the sponsor after you withdraw your permission.

Your rights to privacy are legally protected by federal and provincial laws that require safeguards to ensure that your privacy is respected.

By signing this form you do not give up any of your legal rights against the investigators, sponsor or involved institutions for compensation, nor does this form relieve the investigators, sponsor or involved institutions of their legal and professional responsibilities.

You will be given a copy of this signed and dated consent form prior to participating in this study.

**Conflict of Interest**

This centre is receiving funds from London Health Sciences Foundation to help offset the costs of conducting this research. Lawson Health Research Institute is a hospital based research institute. The researchers at this centre will not receive any direct benefit for conducting this study.

The doctor treating you also may be the doctor in charge of the study.

If you would like additional information about the funding for this study, or about the role of the doctor in charge of this study, please speak to the study staff or to the Office of the Chair of the Ontario Cancer Research Ethics Board. (Contact information below).

**Contacts**

If you have questions about taking part in this study, or if you suffer a research-related injury, you should talk to your study doctor. Or, you can meet with the doctor who is in charge of the study at this institution. That person is:

| *Name* |  | *Telephone #* |
| --- | --- | --- |

| Telephone: 416-673-6648 |  | Toll Free: 1-866-678-6427 ext. 6648 |
| --- | --- | --- |

If you have questions about your rights as a participant or about ethical issues related to this study, you can talk to someone who is not involved in the study at all. Please contact the Office of the Chair of the Ontario Cancer Research Ethics Board at:

**Signatures**

- All of my questions have been answered,
- I understand the information within this informed consent form,
- I allow access to my medical records and specimens as explained in this consent form,
- I am aware of the risks to me of participating in the study and the risks to the fetus if I become pregnant or father a child during this study,
- I do not give up any of my legal rights by signing this consent form,
- I agree to take part in this study.

| Signature of Participant |  | Printed Name |  | Date |
| --- | --- | --- | --- | --- |

| Signature of Person Conducting the Consent Discussion |  | Printed Name |  | Date |
| --- | --- | --- | --- | --- |

**Participant Assistance**

**Complete the following declaration only if the participant is unable to read:**

- The informed consent form was accurately explained to, and apparently understood by, the participant, and
- Informed consent was freely given by the participant

| Signature of Impartial Witness |  | Printed Name |  | Date |
| --- | --- | --- | --- | --- |

**Complete the following declaration only if the participant has limited proficiency in the language in which the consent form is written and interpretation was provided as follows:**

- The informed consent discussion was interpreted by an interpreter, and
- A sight translation of this document was provided by the interpreter as directed by the research staff conducting the consent.

**Interpreter Declaration and Signature:**

By signing the consent form I attest that I provided a faithful interpretation for any discussion that took place in my presence, and provided a sight translation of this document as directed by the research staff conducting the consent.

| Signature of Interpreter |  | Printed Name |  | Date |
| --- | --- | --- | --- | --- |
